# Ethnopharmacological insights into uterine fibroids: a review of etiology, and therapeutic potential of natural products

**DOI:** 10.3389/fphar.2025.1714256

**Published:** 2026-01-08

**Authors:** Gcobisa Valencia Manzane, Joe Sekomeng Modise, Bamide Joseph Okoli, Fanyana Mtunzi, Mzimkhulu Ephraim Monapathi

**Affiliations:** 1 Department of Natural Sciences, Vaal University of Technology, Vanderbijlpark, South Africa; 2 Department of Biochemistry, Bingham University, Karu, Nassarawa, Nigeria; 3 Institute of Chemical and Biotechnology, Vaal University of Technology, Southern Gauteng, Science and Technology Park, Sebokeng, South Africa

**Keywords:** African medicinal plants, Albizia tanganyicensis, ethnopharmacology, Gunnera perpensa, phytochemicals, uterine fibroids

## Abstract

Uterine fibroids (leiomyomas) are the most common benign gynecologic tumors, affecting up to 80% of women by age 50, with higher prevalence and symptom severity reported in women of African descent. These monoclonal tumors originate from smooth muscle cells of the myometrium and are classified based on anatomical location (intramural, submucosal, subserosal, or transmural). Clinical manifestations include abnormal uterine bleeding, pelvic pain or pressure, and infertility. The pathogenesis of fibroids is multifactorial, involving hormonal dysregulation (particularly estrogen and progesterone), MED12 gene mutations, extracellular matrix accumulation, and modifiable risk factors such as vitamin D deficiency and obesity. This review synthesizes current knowledge on fibroid etiology, and treatment strategies, with specific focus on the ethnopharmacological relevance of botanical drugs and natural products. Conventional therapies, including surgery and hormone-based medications, are effective but often associated with high cost, side effects, or loss of fertility. In contrast, natural therapies such as vitamins (D, E), epigallocatechin gallate (EGCG), and plant-based formulations offer promising but underexplored alternatives. Special emphasis is placed on Gunnera perpensa L. [Gunneraceae; Gunnerae perpensae radix] and Albizia tanganyicensis Baker [Fabaceae; Albiziae cortex], two species used in South African traditional medicine for gynecological disorders. Ethnobotanical use, phytochemical profiles, and pharmacological activities including anti-inflammatory, antioxidant, anti-proliferative, and uterotonic properties are critically reviewed. Botanical names have been validated, and data were assessed using the Four Pillars of Best Practice in Ethnopharmacology, the ConPhyMP framework, and the GA checklist for reproducibility and quality assurance. By bridging traditional knowledge with current scientific evidence, this review supports the potential role of culturally rooted botanical drugs in integrative fibroid management and highlights directions for future pharmacological and clinical research.

## 1 Introduction

Uterine fibroids (UFs), also known as leiomyomas or myomas, are the most common benign tumors of the female reproductive tract, affecting up to 70%–80% of women by age 50. While often asymptomatic, they can lead to heavy menstrual bleeding, pelvic pain, urinary frequency, and infertility (Guo and Segars, 2012; Akhter et al., 2015). The clinical burden is especially pronounced among African and African-descendant populations, who tend to develop fibroids earlier and with more severe symptoms than other groups (Ghant et al., 2016; Sefah et al., 2023). Despite their prevalence and impact on quality of life, fibroids are frequently underdiagnosed and mismanaged, particularly in low-resource settings.

Current management options include surgery (e.g., myomectomy, hysterectomy), medical therapies (e.g., GnRH analogs, hormonal therapy), and minimally invasive procedures. However, these approaches are often limited by recurrence, side effects, high cost, or loss of fertility (Guo and Segars, 2012; Li et al., 2020). Such limitations have driven interest in complementary and alternative approaches, especially medicinal plants. Across many African cultures, traditional healers have long relied on herbal remedies to manage gynecological conditions, including fibroids. Yet, despite widespread use, evidence on their safety, efficacy, and mechanisms of action remains fragmented and insufficiently validated (Dalton-Brewer, 2016; Ciebiera et al., 2020).

An ethnopharmacological perspective offers a pathway to bridge traditional knowledge with scientific research by documenting plant-based therapies, identifying their bioactive compounds, and aligning them with pharmacological evidence (Nantinda et al., 2025).

This review consolidates current knowledge on uterine fibroids, covering their etiology, risk factors, and treatment strategies. It places special emphasis on ethnomedicinal plants traditionally used against uterine fibroids, critically evaluating their phytochemistry, anti-fibrotic mechanisms, and therapeutic promise.

## 2 Literature search strategy and study selection process

A structured literature search was conducted to identify peer-reviewed studies relevant to uterine fibroids, medicinal plants, phytochemicals, dietary supplements, and ethnopharmacological interventions. The search covered articles published between 2001 and 2025, across the following electronic databases: EBSCOhost, ScienceDirect, Web of Science, Google Scholar, and Sabinet. Boolean operators and keyword combinations were used, including: “uterine fibroids” OR “leiomyoma” AND “natural products” OR “medicinal plants” OR “phytochemicals” OR “ethnopharmacology” AND “anti-inflammation” OR “anti-oxidant.” Reference lists of relevant articles were also screened to identify additional studies not captured in the database search.

Inclusion criteria were: (1) studies reporting medicinal plants, natural products, or phytochemicals with relevance to uterine fibroid pathophysiology, including anti-inflammatory, antioxidant, hormonal-modulating, or anti-proliferative mechanisms; (2) ethnobotanical or ethnopharmacological studies documenting traditional plant use for gynecological or reproductive disorders; (3) studies that provide mechanistic insights or biological evidence supporting the role of natural products in fibroid management.

Exclusion criteria included: (1) clinical case reports or anecdotal reports lacking scientific rigor, mechanistic explanation, or broader applicability; (2) studies not directly related to uterine fibroids, reproductive health, or gynecological conditions; (3) studies focusing exclusively on male reproductive disorders or unrelated disease models.

**FIGURE 1:**
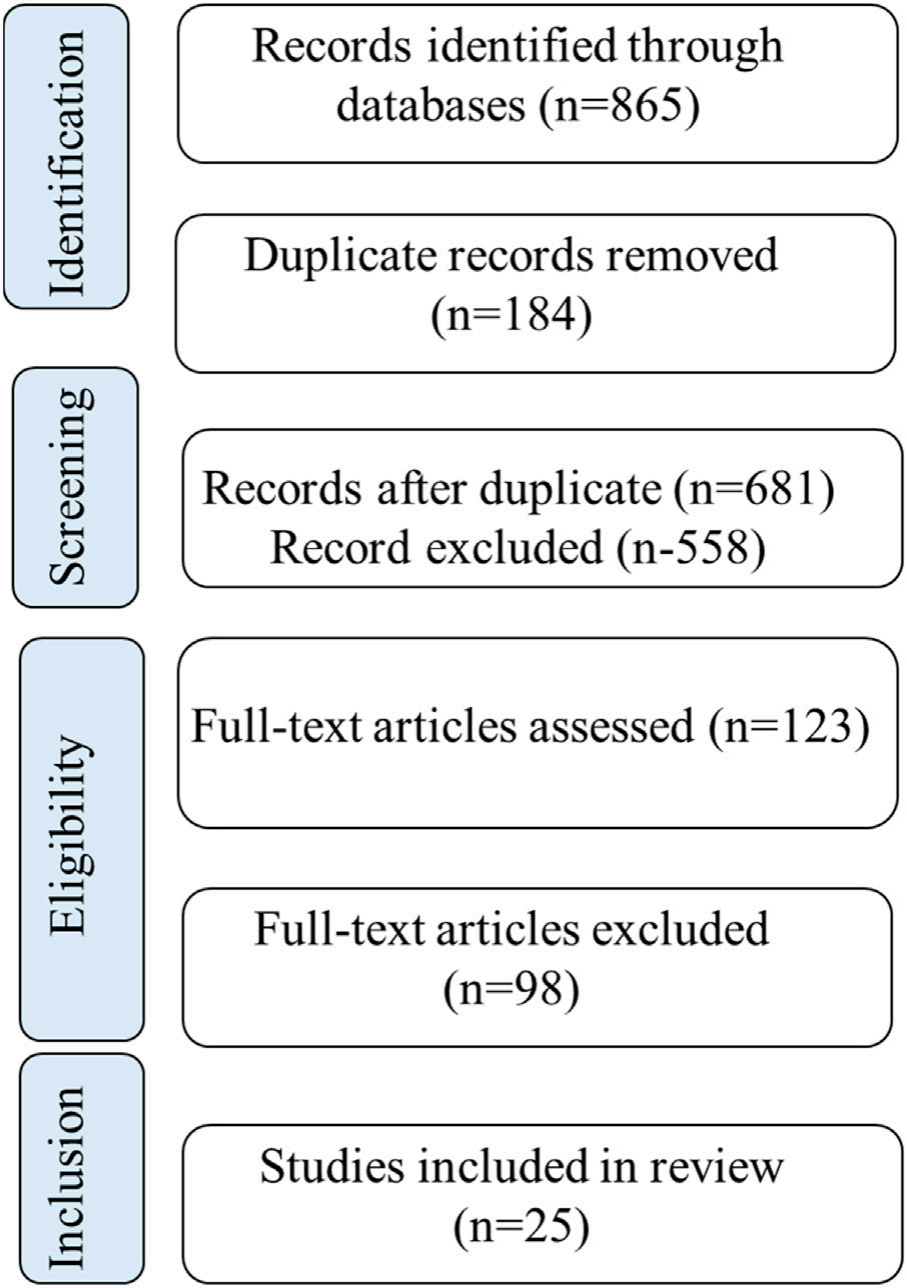
PRISMA flow diagram for the identification and selection of studies on uterine fibroids and medicinal plants.

To ensure methodological rigor and transparency, the review adhered to the Preferred Reporting Items for Systematic Reviews and Meta-Analyses (PRISMA) guidelines. A structured workflow was followed, beginning with the identification of studies from multiple databases, removal of duplicates, and subsequent title and abstract screening. Full-text articles were then assessed for eligibility according to predefined inclusion and exclusion criteria. Reasons for exclusion at each stage were documented to maintain transparency. The overall process of study identification, screening, eligibility assessment, and final inclusion is illustrated in the PRISMA-style flow diagram (Figure 1). This systematic approach ensured that only scientifically robust and thematically relevant literature was incorporated into the final synthesis.

## 3 Pathophysiology and etiology of uterine fibroids

**FIGURE 2:**
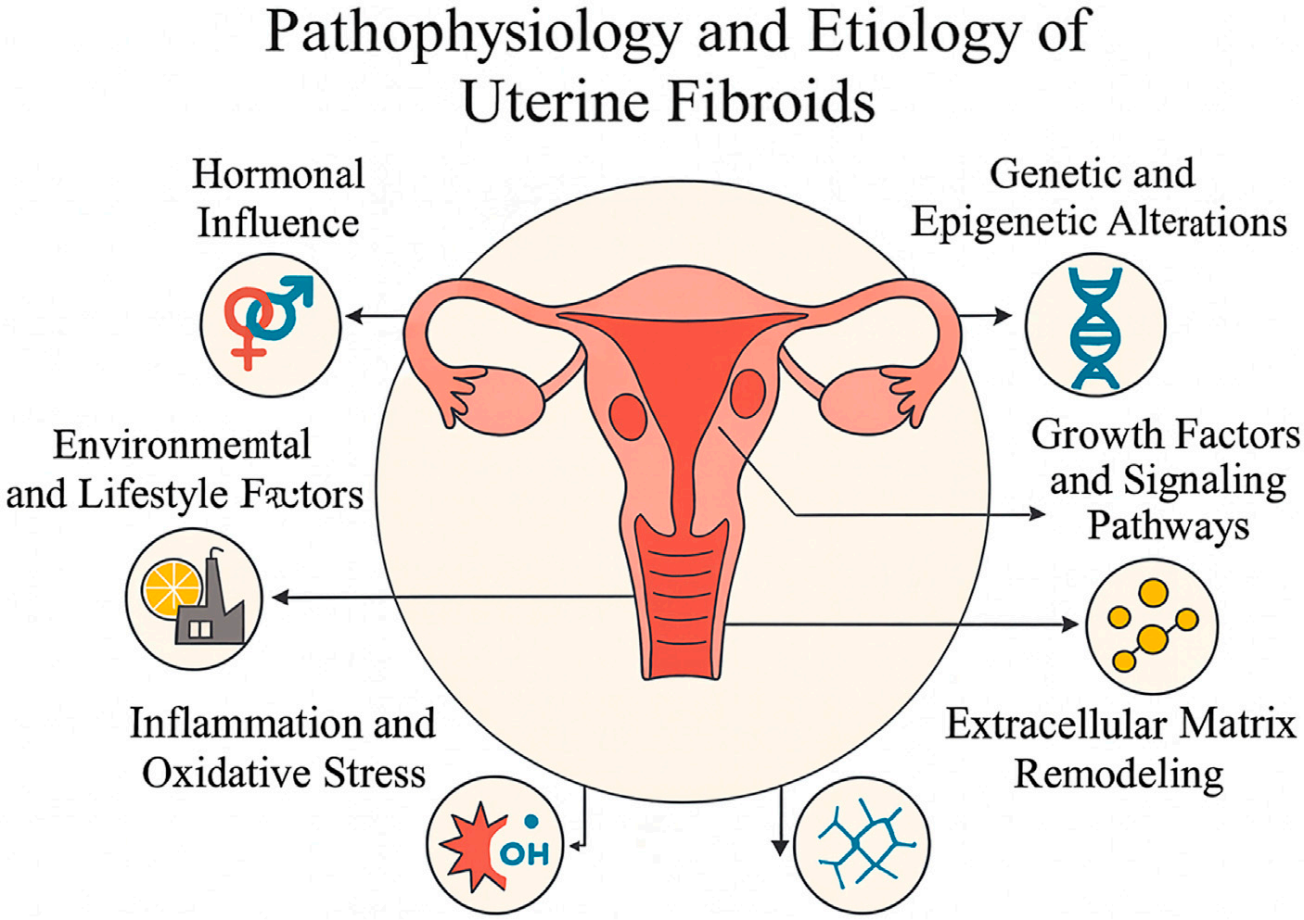
Conceptual overview of the pathophysiology and etiology of uterine fibroids.

The etiology of uterine fibroids is complex and multifactorial, involving the interplay of hormonal, genetic, molecular, inflammatory, and environmental factors. These mechanisms collectively contribute to abnormal smooth muscle cell proliferation and excessive extracellular matrix (ECM) deposition, resulting in fibroid formation and progression (Figure 2).

### 3.1 Hormonal influence: estrogen and progesterone

Estrogen and progesterone are central to fibroid pathogenesis. These sex steroids regulate uterine growth and are often elevated in women with fibroids. Estrogen promotes cellular proliferation, while progesterone contributes to fibroid growth by enhancing extracellular matrix (ECM) production and suppressing apoptosis. Imbalances in these hormones, such as prolonged estrogen dominance, can disrupt normal uterine physiology and drive fibroid formation (Flake et al., 2003; Reis et al., 2016; Ali et al., 2023).

### 3.2 Genetic and epigenetic alterations

Uterine fibroids are associated with a range of genetic and epigenetic changes that contribute to their development and heterogeneity. One of the most frequently reported somatic mutations occurs in the mediator complex subunit 12 (MED12) gene, identified in approximately 70% of fibroid cases. These mutations disrupt transcriptional regulation and cell cycle control, leading to abnormal proliferation of uterine smooth muscle cells (Moravek and Bulun, 2015). Additionally, chromosomal rearrangements such as translocations involving chromosomes 12 and 14, and deletions in specific loci have been observed. Epigenetic mechanisms, including deoxyribonucleic acid (DNA) methylation, histone modifications, and microRNA (miRNA) dysregulation, also play critical roles by altering gene expression without changing the DNA sequence. These changes can silence tumor suppressor genes or activate proliferative and fibrotic pathways, further contributing to fibroid progression.

### 3.3 Growth factors and signaling pathways

Uterine fibroids are characterized by overexpression of several growth factors, including insulin-like growth factors (IGFs), transforming growth factor-beta (TGF-β), epidermal growth factor (EGF), and platelet-derived growth factor (PDGF). These growth factors promote fibroid development by driving cellular proliferation (mitogenesis) and supporting the formation of new blood vessels (angiogenesis). Among them, IGFs are particularly significant, as they interact with estrogen and insulin signaling pathways to amplify fibroid cell growth and proliferation (Baird et al., 2009; Ciarmela et al., 2011).

### 3.4 Extracellular matrix (ECM) remodeling

Uterine fibroids are characterized by excessive and disorganized extracellular matrix accumulation, primarily composed of collagen, fibronectin, laminin, and proteoglycans. This leads to tissue stiffening and altered biomechanical properties, which activate pathways that promote fibroid cell survival and proliferation, while also making them more resistant to programmed cell death (apoptosis). The imbalance between matrix metalloproteinases (MMPs) and their inhibitors, tissue inhibitors of metalloproteinases (TIMPs) also contributes to abnormal ECM remodeling in fibroids (Zigalobari and Alexandrivna, 2020).

### 3.5 Inflammation and oxidative stress

Uterine fibroids are associated with disrupted antioxidant defense systems, resulting in imbalances between reactive oxygen species (ROS) and antioxidant activity. This oxidative stress leads to cellular damage, including DNA harm and impaired cellular function. ROS, such as superoxide and hydroxyl radicals, are by-products of normal cellular metabolism but become harmful when produced in excess. Additionally, fibroids are linked to chronic low-grade inflammation, characterized by elevated levels of pro-inflammatory cytokines (e.g., tumor necrosis factor alpha [TNF-α] and interleukin-6 [IL-6]). These inflammatory mediators not only enhance ROS production but also activate signaling pathways that support fibroid development and progression. The combined effects of oxidative stress and inflammation contribute to fibrotic changes within the uterus, including overproduction of extracellular matrix proteins and tissue thickening, which collectively promote fibroid formation and expansion (AlAshqar et al., 2021; Ishikawa et al., 2024).

### 3.6 Environmental and lifestyle factors

Environmental and lifestyle factors significantly impact uterine fibroid development and progression. Endocrine-disrupting chemicals (EDCs) like phthalates, polychlorinated biphenyls (PCBs), and bisphenol A (BPA), found in everyday products, can interfere with hormone signaling, potentially promoting fibroid growth by mimicking estrogen and affecting hormonal pathways. Obesity, vitamin D deficiency, early menarche, lack of physical activity, and high-fat diets are also linked to increased fibroid risk and severity. Specifically, obesity elevates estrogen levels, while vitamin D deficiency has been correlated with larger and more symptomatic fibroids, due to vitamin D’s anti-fibrotic, anti-inflammatory, and anti-proliferative effects (Giuliani et al., 2020; Yang et al., 2022).

## 4 Conventional treatment strategies for uterine fibroids

This review aligns with the Sustainable Development Goals (SDGs) established in 2015 by United Nations member states to create global partnerships among all countries (UNDP, 2015). According to SDG 3, prevention and treatment of communicable and non-communicable disease epidemics should end or be prevented by 2030. Effective and successful treatment of uterine fibroids champions SDG 3 with emphasis on good health and wellbeing for all. The choice of treatment for uterine fibroids largely depends on size, number, growth rate, location, and symptoms (Donnez and Dolmans, 2016). Current treatment modalities fall into two major categories: surgical interventions and medical (pharmacological) therapies.

### 4.1 Surgical treatment

Surgical management is often considered the most definitive approach, particularly for women with large or symptomatic fibroids (Ciebiera et al., 2020). Common surgical strategies include:

Hysterectomy involves removal of the entire uterus along with fibroids. This treatment option offers complete symptom resolution and is suitable for women who do not wish to preserve fertility (De La Cruz and Buchanan, 2017). However, myomectomy removes fibroids while preserving the uterus. It is the preferred treatment for women who wish to maintain their uterus and preserve fertility (Guo and Segars, 2012). Conversely, myomectomy carries risks of fibroid recurrence and surgical complications.

Magnetic Resonance-guided Focused Ultrasound Surgery (MRgFUS) is a non-invasive procedure that uses focused ultrasound waves to heat and destroy fibroids (Donnez and Dolmans, 2016). It is mostly applied in treating small to medium-sized fibroids, typically less than 10 cm in diameter (Ciebiera and Łoziński, 2020). Uterine Artery Embolization (UAE) is a minimally invasive procedure that involves blocking the blood vessels that supply blood to the fibroids (Gupta et al., 2014). Uterine Artery Embolization (UAE) is a minimally invasive procedure that involves blocking blood vessels supplying fibroids (Gupta et al., 2014). Ultimately, fibroids shrink and undergo necrosis. Each surgical option carries benefits and limitations related to fertility preservation, recurrence risk, invasiveness, and cost.

### 4.2 Medical (pharmacological) treatments

Medical therapy is generally recommended for women who desire non-invasive options that alleviate fibroid symptoms (Pérez-López et al., 2014). This treatment option does not involve fibroid removal, thus representing a good choice for women desiring future fertility (Taylor and Leppert, 2012). Women with uterine fibroid symptoms should consider medication therapy before undergoing surgery. However, medical therapy is not recommended for large fibroids (Taylor and Leppert, 2012). Non-steroidal Anti-inflammatory Drugs (NSAIDs) are over-the-counter medications used to relieve pain, reduce inflammation, and control heavy bleeding (Bofill Rodriguez et al., 2019). However, they do not address underlying pathology. Tranexamic is an antifibrinolytic agent used to manage menorrhagia by preventing clot breakdown. Its effects are temporary, and fibroids often regrow after discontinuation (Leminen and Hurskainen, 2012; De La Cruz and Buchanan, 2017).

Selective Progesterone Receptor Modulators (SPRMs) are a new class of synthetic compounds that have demonstrated therapeutic potential against uterine fibroids, endometriosis, endometrial cancer, and breast cancer (Tristan et al., 2012). SPRMs such as ulipristal acetate block progesterone effects. Ultimately, fibroids shrink and heavy menstrual bleeding is alleviated (Piecak et al., 2017). Hormonal birth control methods such as oral pills, patches, injections, and hormonal intra-uterine devices (IUDs) contain estrogen and progestin. As mentioned by De La Cruz and Buchanan (2017), contraception may not cure fibroids but provides symptomatic relief and helps manage associated symptom (De La Cruz and Buchanan, 2017). However, some hormonal birth control methods may be accompanied by several side effects including headaches, nausea, elevated blood pressure, and changes in sex drive (Sayed et al., 2011). Gonadotropin-releasing hormones (GnRH) agonists temporarily shrink fibroids and help alleviate symptoms such as heavy menstrual bleeding, pelvic pain, and pressure (Lethaby et al., 2017). GnRH therapy is temporary (De La Cruz and Buchanan, 2017). When discontinued, patients may experience symptom recurrence and fibroid regrowth to almost pre-treatment size. The levonorgestrel-releasing Intrauterine System (LNG-IUS) is a small, T-shaped device inserted into the uterus that releases levonorgestrel into the uterine cavity (Ji et al., 2023). Levonorgestrel-releasing intrauterine devices effectively reduce menstrual blood loss by releasing levonorgestrel directly into the uterine cavity, thereby decreasing menstrual flow and addressing dysfunctional uterine bleeding (Wrona et al., 2017). Although medical treatments are generally more accessible and less invasive, they may be associated with side effects, incomplete symptom relief, and high recurrence rates.

### 4.3 Limitations of conventional therapies

Despite being the current standard of care, conventional therapies for uterine fibroids, including surgical and pharmacological interventions, pose significant limitations. Surgical treatments are often effective in removing fibroids or the entire uterus; however, these procedures come with inherent risks such as bleeding, infection, anesthesia complications, and extended recovery periods (Pritts and Olive, 2012; Centini et al., 2024). Medical therapies are primarily used for symptom management. These treatments may temporarily reduce fibroid size or alleviate heavy menstrual bleeding but are rarely curative. Long-term use is often limited by side effects such as hot flashes, bone density loss, mood changes, and rebound fibroid growth upon cessation (Donnez et al., 2014; Stewart, 2015). Moreover, hormonal therapies may not be suitable for all patients, especially those with hormone-sensitive conditions or contraindications to estrogen/progesterone modulation. Another challenge is the recurrence rate of fibroids after certain surgical interventions like myomectomy, particularly in younger women. Studies suggest recurrence rates as high as 15%–30% within 5 years, potentially necessitating repeated interventions (Kramer et al., 2021). Furthermore, access to advanced diagnostic and treatment options remains a major barrier in low- and middle-income countries (LMICs). Limited healthcare infrastructure, high treatment costs, and disparities in healthcare access mean that many women in resource-constrained settings lack appropriate or timely care. Cultural stigma and fear of surgery may also deter some women from seeking biomedical treatments, pushing them toward alternative or traditional medicine (Baird et al., 2003). These limitations indicate the urgent need for alternative, safe, and affordable treatment options. Ethnopharmacological and botanical-based therapies, often rooted in traditional knowledge systems, may offer complementary or stand-alone benefits with fewer side effects. Integrating validated natural products into modern fibroid management protocols could improve accessibility, cultural relevance, and holistic health outcomes.

## 5 Overview of natural products in fibroid management

Growing interest in complementary and alternative medicines (CAMs) has led to investigation of various natural products and botanicals for uterine fibroid management. These agents are typically accessible, cost-effective, and perceived to have fewer systemic side effects than conventional therapies (van Wyk and Prinsloo, 2020). While preclinical studies provide encouraging evidence of anti-estrogenic, anti-fibrotic, anti-inflammatory, and antioxidant activities (Ciebiera et al., 2020; Szydłowska et al., 2022), clinical validation remains scarce. Most natural products have been evaluated only in small-scale, poorly standardized, or preliminary clinical studies, limiting the reliability and generalizability of findings. This gap is clinically significant, as it restricts their safe integration into treatment guidelines, poses challenges for dosage standardization, and leaves patients vulnerable to unregulated use (Shipkowski et al., 2019; Liang et al., 2025). Therefore, while natural products hold promise in fibroid management, rigorous clinical trials are urgently needed to confirm efficacy, establish safety profiles, and support evidence-based recommendations.

### 5.1 Herbal medicines and traditional systems

#### 5.1.1 Natural medicinal preparations

Herbal formulations, widely used in African, Asian, and Latin American cultures, have shown potential in reducing fibroid size, regulating menstruation, and improving fertility (Li et al., 2020; Coulidiaty et al., 2021). Commonly used herbs include *Vitex agnus-castus*, *Curcuma longa* (turmeric), *Camellia sinensis* (green tea), and *Phyllanthus amarus*. These herbs often contain bioactive constituents such as flavonoids, alkaloids, and saponins with antiproliferative and hormone-modulating properties (Abebe, 2002; Enioutina et al., 2020). However, concerns regarding standardization, quality control, and safety profiling remain unresolved, as these preparations are often not regulated by drug authorities (Li et al., 2020).

#### 5.1.2 Traditional Chinese medicine

Previous studies have shown that Traditional Chinese Medicine (TCM), including green tea and Chinese herbal formulations, slow uterine fibroid growth and alleviate associated symptoms (Chen et al., 2014; Bijarnia et al., 2022). TCM practitioners may use herbal medicines and various mind-body practices such as acupuncture and Tai Chi to treat uterine fibroids (Fears et al., 2020). Several ancient Chinese medicine formulas have therapeutic potential to relieve uterine fibroid symptoms and reduce fibroid size without significant side effects (Li et al., 2020).

#### 5.1.3 Phytocompounds–acupuncture therapy in uterine fibroid management

Phytocompounds–acupuncture therapy is an emerging integrative approach proposed for uterine fibroid management, combining the biochemical effects of plant-derived compounds with the neuroendocrine and analgesic benefits of acupuncture. While phytochemicals such as curcumin, epigallocatechin-3-gallate (EGCG), resveratrol, and indole-3-carbinol, etc., have demonstrated anti-estrogenic, anti-proliferative, and antioxidant activities, acupuncture is hypothesized to modulate the hypothalamic–pituitary–ovarian (HPO) axis, regulate reproductive hormones, reduce pelvic inflammation, and alleviate pain through central neurotransmitter pathways. However, the mechanism by which acupuncture could directly influence fibroid growth remains vague and poorly defined, with current evidence largely limited to symptom relief rather than tumor modification. No controlled studies have validated the combined effect of phytocompounds and acupuncture, making this a promising yet underexplored area that requires rigorous mechanistic and clinical research (Olowosoke and Chukwuemeka, 2025).

### 5.2 Dietary supplements and micronutrients

#### 5.2.1 Dietary supplements

Dietary supplements are products consumed with meals or at specific intervals to supplement normal diet (Hassan et al., 2020). They are largely added to improve overall health, fulfill nutritional gaps, and address specific health goals. Examples of dietary supplements are products containing vitamins and minerals, or other substances with nutritional or physiological effects such as amino acids, essential fatty acids, probiotics, plants, and herbal extracts. According to studies conducted by Martin et al. (2011) and Szydłowska et al. (2022), intake of certain dietary supplements could contribute to uterine fibroid treatment (Martin et al., 2011; Szydłowska et al., 2022).

#### 5.2.2 Vitamins

Vitamin A (retinol) regulates cell growth and differentiation (Szydłowska et al., 2022). Studies conducted by Malik et al. (2009) and Zaitseva et al. (2008) confirmed that retinoids inhibit the growth of primary cultures of human uterine Tumors (Zaitseva et al., 2008; Malik et al., 2009).

Vitamin B6 (pyridoxine) has been shown to exhibit anti-inflammatory properties (Mikkelsen et al., 2023). Chronic inflammation in uterine tissue contributes to uterine fibroid development and growth by promoting cell proliferation (Ciebiera et al., 2021). Vitamin B6 slows fibroid growth by reducing inflammation in uterine tissues. Vitamin B12 (Cobalamin) plays essential roles in red blood cell formation (Karmi et al., 2011). This function is important in managing anemia resulting from heavy menstrual bleeding associated with uterine fibroids. Vitamins C (Ascorbic acid) and E (Tocopherol) slow fibroid growth through their anti-inflammatory and antioxidant properties (Chambial et al., 2013). In studies conducted by Al-Hendy and Badr (2014) and Hajhashemi et al. (2019), administration of vitamin D3 reduced uterine fibroid size (Al-Hendy and Badr, 2014; Hajhashemi et al., 2019). Antioxidants protect uterine cells from oxidative damage caused by free radicals (highly reactive and unstable molecules) produced in the body (Nath and Roy, 2021). Neutralization of harmful free radicals by antioxidants prevents them from causing cellular damage while slowing fibroid growth.

#### 5.2.3 Minerals

Uterine fibroids may cause heavy menstrual flow in some patients, potentially resulting in anemia (Sriprasert et al., 2017). Intake of iron supplements helps address iron deficiency caused by heavy bleeding (Mantadakis et al., 2020). Magnesium, an essential electrolyte, regulates muscle contraction and relaxation (Batta, 2017). Magnesium supplements may alleviate uterine fibroid symptoms by reducing muscle cramps and spasms in the uterus (Gutman et al., 2022). Selenium and Zinc are essential micronutrients with potent antioxidant and anti-inflammatory properties (Muzembo et al., 2015; Grüngreiff et al., 2016). They play pivotal roles in uterine fibroid treatment by managing oxidative stress and inflammation associated with fibroid development and growth.

### 5.3 Plant-derived antioxidants and phytochemicals

Numerous dietary antioxidants and plant-derived bioactive compounds have demonstrated significant potential in prevention and management of uterine fibroids. Previously discussed micronutrients such as vitamins A, C, D, and E, as well as essential minerals like selenium and zinc, have shown anti-inflammatory and antioxidative effects. In addition to these nutrients, specific phytochemicals, especially polyphenols, flavonoids, and indoles, exhibit anti-fibrotic, anti-estrogenic, and anti-inflammatory properties, contributing to their therapeutic potential.

Among these, epigallocatechin gallate (EGCG), a key catechin found in green tea, has attracted considerable attention. EGCG is a potent antioxidant that inhibits proliferation and induces apoptosis in uterine fibroid cells, as demonstrated in vitro studies (Islam et al., 2014; Zhang et al., 2014; Szydłowska et al., 2022). The anti-fibrotic effects of green tea polyphenols are largely attributed to their capacity to modulate cell signaling pathways involved in oxidative stress and inflammation. , the bioactive component in turmeric (*C. longa*), suppresses the NF-κB signaling pathway and downregulates anti-apoptotic Bcl-2 family proteins, thereby reducing fibroid cell viability and promoting cell death. Curcumin also exhibits anti-estrogenic properties, contributing to its efficacy in managing estrogen-dependent conditions like fibroids (Szydłowska et al., 2022).

Quercetin, a flavonoid present in onions, apples, and various fruits, along with Indole-3-carbinol (I3C) found in cruciferous vegetables such as broccoli and cabbage, have demonstrated strong anti-fibrotic and anti-proliferative activities. These compounds inhibit pathways associated with extracellular matrix remodeling, fibroid cell migration, and fibrogenesis. Greco et al. (2020) and AlAshqar et al. (2023) showed that quercetin and I3C not only reduce fibroid growth but also disrupt cell motility, which may help prevent progression and symptom severity (Greco et al., 2020; AlAshqar et al., 2023). Collectively, these natural compounds may offer complementary or alternative strategies to conventional therapies by targeting oxidative and hormonal mechanisms involved in fibroid pathogenesis.

### 5.4 Computational and AI-assisted in silico bioprospecting for uterine fibroid therapeutics

Recent advances in computational pharmacology have introduced powerful AI-assisted tools for bioprospecting natural products in uterine fibroid (UF) management, expanding beyond traditional *in vitro* ethnopharmacology approaches. Following the identification of hundreds of previously uncharacterized UF-associated genes, *in silico* strategies, including molecular docking, network pharmacology, absorption, distribution, metabolism, excretion and toxicity (ADMET) prediction, quantitative structure–activity relationship (QSAR) modelling, and machine learning–based target prediction, are increasingly being used to link phytocompounds with specific genetic and molecular pathways involved in UF pathogenesis.

Recent computational studies across pharmacology, bioinformatics, and structural biology platforms have demonstrated that compounds such as epigallocatechin gallate (EGCG), curcumin, quercetin, kaempferol, and indole-3-carbinol exhibit strong predicted binding affinities and multi-target interactions with key UF-related pathways, including transforming growth factor-beta/Smad (TGF-β/Smad) signaling, mitogen-activated protein kinase (MAPK), and nuclear factor kappa-light-chain-enhancer of activated B cells (NF-κB) pathways, matrix metalloproteinases (MMPs), extracellular matrix regulators, and hormone-responsive genes (Tiwari et al., 2023; Buyukcelebi et al., 2024; Olowosoke et al., 2024; 2025). These AI-driven predictions highlight the polypharmacological nature of plant-derived compounds and their alignment with the multifactorial pathophysiology of UF. Nevertheless, rigorous experimental validation remains essential before these computational insights can be translated into evidence-based therapeutic development.

### 5.5 Research gaps and future directions

Despite encouraging findings, clinical validation of most natural agents remains limited. Small sample sizes, lack of standardized dosing, and short intervention durations restrict generalizability. Moreover, potential herb-drug interactions, poor bioavailability, and variation in product composition warrant further investigation. Nonetheless, with advancing tools in ethnopharmacology, molecular pharmacology, and clinical trials, natural products offer promising adjuncts or alternatives to conventional therapies, especially in settings with limited access to biomedical care.

### 5.6 Barriers to clinical translation of natural products

Despite extensive preclinical research, many natural products with promising biological activities have not progressed to effective clinical therapies for uterine fibroids or related gynecological disorders. This translational gap is largely driven by variability in plant material due to inconsistent harvesting, processing, and storage, which compromises reproducibility; incomplete phytochemical characterization, making it difficult to identify active principles and standardize dosages; and lack of comprehensive pharmacokinetic and toxicological data required to establish safety profiles (Shipkowski et al., 2019). Furthermore, combinations of factors including complex and incompletely understood pathophysiology of fibroids, limitations in translating *in-vitro* and animal studies to human physiology, and absence of high-quality, large-scale human clinical trials to validate findings for natural compounds contribute to this gap (Yang et al., 2022). Regulatory hurdles and limited funding further hinder development of botanical medicines compared with synthetic drugs. Recognizing these challenges is critical when evaluating *Gunnera perpensa* and *Albizia tanganyicensis*; while their traditional use and preclinical activities are encouraging, translation into viable therapeutic options will require standardized extraction methods, rigorous mechanistic investigations, and carefully designed clinical studies. A summary of the major natural compounds investigated in ethnomedicinal plants for uterine fibroids, along with their reported biological activities, sources and their relevance to uterine fibroids, is presented in Table 1.

**TABLE 1:** Summary of key natural compounds in ethnomedicinal plants for uterine fibroids.

Compound	Phytochemical class	Biological activity	Source plant(s)	Relevance to uterine fibroids	Reference(s)
Quercetin	Flavonoid	Antioxidant, anti-inflammatory, antiproliferative	Allium cepa, Gunnera perpensa	Suppresses extracellular matrix deposition and inhibits migration and proliferation of leiomyoma cells	Greco et al. (2020)
Curcumin	Polyphenol	Antioxidant, anti-inflammatory, pro-apoptotic	Curcuma longa	Inhibits fibroid cell proliferation and modulates estrogen/progesterone receptor expression	Islam et al. (2014)
Genistein	Isoflavone	Phytoestrogenic, anti-inflammatory	Glycine max (soybean)	Modulates estrogen receptor activity and suppresses fibroid cell growth	Islam et al. (2014)
EGCG (Epigallocatechin gallate)	Catechin (Flavonoid)	Antioxidant, anti-angiogenic, antiproliferative	Camellia sinensis (Green tea)	Inhibits fibroid cell growth, collagen production and angiogenesis	Islam et al. (2023) and Szydłowska et al. (2022)
Resveratrol	Stilbene	Antioxidant, anti-inflammatory, antiproliferative	Vitis vinifera, Polygonum cuspidatum	Reduces extracellular matrix accumulation and fibrotic markers in leiomyoma tissue	Islam et al. (2014)
Berberine	Alkaloid	Anti-inflammatory, antioxidant, antifibrotic	Berberis spp., Coptis chinensis	Inhibits TGF-β signalling and ECM production under fibrotic conditions	Wang et al. (2024)
Apigenin	Flavone	Anti-inflammatory, anti-estrogenic	Chamomile, Parsley	Downregulates hormone receptors and inhibits cell cycle progression	Darabi et al. (2020)
Kaempferol	Flavonoid	Antioxidant, antifibrotic	Gunnera perpensa, Ginkgo biloba	Suppresses fibroid cell proliferation; downregulates ER, IGF-1 and VEGF signalling	Li et al. (2016)
Lupeol	Triterpenoid	Anti-inflammatory, antitumor	Albizia spp., Mangifera indica	Inhibits fibroblast proliferation and induces apoptosis	Lucetti et al. (2010)
β-Sitosterol	Phytosterol	Anti-inflammatory, hormone-modulating	Albizia spp., pumpkin seed, saw palmetto	Modulates estrogenic signalling and reduces fibrotic progression	Wen et al. (2023)

## 6 Ethnopharmacological review of medicinal plants used against uterine fibroids

### 6.1 Selection of medicinal plants

The selection of medicinal plants for this review was informed by oral testimonies from five experienced traditional health practitioners (THPs) representing communities in Gauteng, Eastern Cape, and KwaZulu-Natal, South Africa. These practitioners, who have long-standing experience in managing women’s reproductive health conditions, independently identified species used for gynecological disorders such as abnormal uterine bleeding, menstrual pain, and symptoms associated with uterine fibroids. Consensus was reached through repeated mentions across practitioners, with species cited by all healers prioritized for inclusion. This approach provided a culturally authentic yet systematic and reproducible framework for identifying plants of interest.

In this process, *G. perpensa* and *A. tanganyicensis* consistently emerged as key species in herbal formulations for fibroid-related complaints. Both plants are widely recognized in South African traditional medicine for reproductive health, particularly in alleviating heavy menstrual bleeding, pelvic discomfort, and hormone-related symptoms.

Traditional health practitioners, also known as traditional healers, indigenous healers, or folk healers, are custodians of ancestral medical knowledge passed down through oral tradition (Ozioma and Chinwe, 2019). While such knowledge risks being lost with successive generations, it has been increasingly documented in ethnobotanical texts and global herbals (Chin et al., 2006). These practitioners possess invaluable knowledge of local flora and traditional healing practices, making them essential resources in the search for new medicinal plant species and potential therapeutic applications (Moges and Moges, 2020). They also provide insight into preparation methods, dosage forms, and synergistic combinations with other plant materials (Sobiecki, 2014). Importantly, traditional medicine practices in South Africa often rely on complex herbal mixtures rather than single-plant remedies. These combinations typically include different plant species or parts, designed to enhance efficacy through synergistic interactions (Ndhlala et al., 2011). To ensure relevance and sustainability, plant selection was further guided by: (i) cultural significance and continuity of use within indigenous healthcare systems; (ii) local availability to support ongoing traditional and scientific applications; and (iii) preliminary pharmacological evidence of anti-inflammatory, antioxidant, and hormone-modulating effects relevant to fibroid pathophysiology. This combination of oral consensus and scientific rationale provides a culturally grounded yet evidence-oriented basis for further investigation into the therapeutic potential of these species.

#### 6.1.1 Traditional preparation and formulation

According to the consulted traditional health practitioners (THPs), uterine fibroid preparations commonly involve a standardized herbal mixture of Albizia tanganyicensis stem bark and Gunnera perpensa leaves. Specifically, three teaspoons of powdered *A. tanganyicensis* stem bark are mixed with one teaspoon of powdered *G. perpensa* leaves to form a dry herbal blend. From this mixture, one teaspoon is used to prepare approximately 500 mL of a decoction by boiling in water. After reaching a lukewarm temperature of about 40 °C, ethanol is added to enhance extraction efficiency and act as a natural preservative. The mixture is then filtered, allowed to cool, and stored under refrigeration. This formulation reflects traditional principles aimed at maximizing therapeutic potency while ensuring stability and shelf life.

#### 6.1.2 Solvent systems and dosage practices reported by THPs

Traditional healers primarily employ a water-ethanol solvent system for the extraction of bioactive compounds from the herbal mixture. Water serves as the primary solvent during decoction, facilitating the extraction of polar constituents such as phenolics and flavonoids, while ethanol is added post-heating to enhance the extraction of moderately polar and non-polar compounds and to improve preservation. Following filtration and cooling, the herbal extract is stored in refrigerated conditions to maintain stability and prevent microbial growth. The standardized traditional dosage involves oral administration of 10 mL of the decoction three times daily, typically prescribed over a period depending on symptom severity, patient response, and traditional diagnostic assessment.

### 6.2 Botanical and taxonomic validation

The botanical identities of the medicinal plants used in this study were primarily determined through traditional health practitioner (THP) expertise, based on morphological characteristics, therapeutic knowledge, and cultural recognition. As custodians of indigenous knowledge systems, THPs have historically identified and used these species for gynecological health, including treatment of uterine fibroids (Alum, 2024). To support accurate botanical documentation and align with scientific standards, plant names were cross-checked and validated using internationally recognized taxonomic databases, including Plants of the World Online (POWO), World Flora Online (WFO), and the Medicinal Plant Names Services (MPNS). This ensured consistency in nomenclature, family classification, and pharmacopeial references where applicable (Yao et al., 2021).

The following taxonomic identifications were confirmed: *G. perpensa* L. [Gunneraceae; Gunnerae perpensae radix] and *A. tanganyicensis* Baker [Fabaceae; Albiziae cortex] (Berger, 2001; Maroyi, 2016). Although no formal herbarium authentication (e.g., through SANBI) was conducted, plant samples were identified by experienced traditional healers with long-standing practical knowledge of these species. This practice aligns with ethnopharmacological approaches that recognize traditional identification methods as valid, particularly when supported by consistent scientific taxonomy.

### 6.3 Phytochemical composition

Phytochemical investigations of the selected medicinal plants have revealed the presence of diverse bioactive compounds associated with therapeutic effects, particularly in relation to inflammation, oxidative stress, and hormonal modulation. Previous studies on *G. perpensa* L. have documented a rich profile of secondary metabolites. According to Simelane et al. (2010), this species contains phenolic compounds, tannins, cardiac glycosides, flavonoids, terpenoids, and saponins (Simelane et al., 2010). Additional phytochemical screening conducted in the present study confirmed the presence of flavonoids, alkaloids, phenolics, tannins, and terpenoids in *G. perpensa*. These classes of compounds are well known for their antioxidant and anti-inflammatory properties, which support the traditional use of the plant in managing gynecological conditions, including uterine fibroids.

In the case of *A. tanganyicensis* Baker, there is limited published data on its phytochemistry. Therefore, the current study contributes new findings by reporting that crude extracts of *A. tanganyicensis* contain steroids but lack alkaloids. These results were consistent across both acetone and ethyl acetate extracts. The absence of alkaloids and presence of steroidal compounds suggest a distinct phytochemical profile, which may account for some of the plant’s traditional therapeutic effects. It is important to note that the distribution and concentration of pharmacologically active metabolites in both species vary depending on the plant part used and the solvent employed for extraction. This demonstrates the importance of solvent selection and extraction conditions in optimizing the yield of bioactive compounds for pharmacological analysis.

#### 6.3.1 Influence of preparation and solvent systems on phytochemical composition

The traditional preparation method significantly influences the phytochemical profile of the herbal formulation. The decoction process primarily favors the extraction of water-soluble compounds, such as phenolics, flavonoids, tannins, and glycosides, which are associated with antioxidant and anti-inflammatory activities. The subsequent addition of ethanol expands the extraction spectrum by solubilizing less polar constituents, including terpenoids and steroidal compounds, particularly relevant for *A. tanganyicensis*. Furthermore, the use of moderate temperature (around 40 °C) before ethanol addition may help preserve thermolabile compounds that could degrade under prolonged high heat. Thus, the traditional solvent system and preparation technique likely contribute to the broad phytochemical diversity and potential synergistic bioactivity observed in this formulation.

## 7 Ethnopharmacological background and cultural significance of *Albizia tanganyicensis* (Umphaphama)

*Albizia tanganyicensis*, known locally in the isiNdebele language as *Umphaphama*, holds profound cultural and medicinal legacy among traditional health practitioners (THPs) in South Africa. The name *Umphaphama* originates from the isiNdebele verb *phaphama*, meaning “wake up.” This term metaphorically reflects the plant’s traditional use: to stimulate or awaken dormant emotional and physical intimacy, particularly in women.

In traditional African societies, particularly during the era of arranged marriages, it was not uncommon for newly married women to experience emotional detachment or resistance toward unfamiliar husbands (Bunting and Lawrance, 2021). In such cases, family elders would consult THPs, who would prepare herbal remedies containing *Umphaphama* to gently stimulate affection, receptivity, and sexual arousal in brides. The formulation was said to help “wake up” their emotional and physical connection to new husbands, encouraging acceptance and harmony in relationships.

From a phytochemical perspective, the therapeutic potential of *A. tanganyicensis* is thought to be largely attributed to its steroidal constituents. While scientific studies on this species remain limited, preliminary phytochemical analyses have confirmed the presence of steroid compounds. Steroids are known to influence hormonal regulation, reduce inflammation, and affect libido and reproductive health. Women suffering from inflammation associated with uterine fibroids often endure chronic pain, hormonal imbalance, and suppressed sexual desire (Lorenz, 2019). *Umphaphama*, through its bioactive steroidal profile, may help restore hormonal balance, alleviate pain, and enhance libido. Although these pharmacological effects have yet to be fully validated in *A. tanganyicensis*, its traditional use suggests that its steroidal profile may contribute significantly to symptomatic relief of uterine fibroids.

Given the lack of comprehensive scientific research on *A. tanganyicensis*, this study seeks to bridge the gap between traditional knowledge and pharmacological validation. By characterizing its bioactive compounds and evaluating their biological activities, we aim to support or refute traditional claims surrounding *Umphaphama*, particularly its potential role in managing uterine fibroids and improving women’s reproductive wellbeing.

### 7.1 Dosage modulation in clinical ethnopharmacology

In clinical ethnopharmacology, understanding and applying traditional dosage practices is essential for preserving efficacy while minimizing adverse effects (Rodrigues and Barnes, 2013). Traditional Health Practitioners often adjust dosages based on age, sex, physiological state (e.g., pregnancy or sexual inactivity), and desired therapeutic outcomes (Pace and Schwarz, 2012). In the case of *Umphaphama*, dosage modulation is used intentionally: for married women experiencing fibroid-related symptoms and reduced libido, a standard or slightly elevated dosage may be prescribed to stimulate hormonal and emotional responses. Conversely, for single or celibate women, dosage is deliberately lowered to avoid excessive stimulation, which could lead to discomfort or psychological distress. This culturally sensitive dosage adjustment showcases the deep experiential knowledge of THPs, emphasizing the importance of context-aware phytotherapy.

This individualized approach resonates with modern clinical pharmacology principles, where dose–response relationships and personalized medicine are central. However, specific dosage ranges and standardized preparation methods for *A. tanganyicensis* and *G. perpensa* remain largely undocumented in scientific literature. Ethnopharmacologists therefore face the dual task of respecting indigenous dosage frameworks while translating them into measurable parameters that can be validated experimentally. Future work should prioritize pharmacological dose–response studies, coupled with toxicological assessments, to establish safe therapeutic ranges and clarify mechanisms of action against uterine fibroids.

### 7.2 Pharmacological activities

Numerous studies conducted in South Africa have investigated the pharmacological activities of *G. perpensa*, revealing a wide range of bioactive properties. These include anti-inflammatory and antinociceptive effects (Nkomo and Kambizi, 2009), antioxidant activity (Simelane et al., 2010), uterine contractile and uterotonic effects (Kaido et al., 1997; Khan et al., 2004), antimicrobial activity (Nkomo and Kambizi, 2009), and antitumor potential (Maroyi, 2016; Mathibe et al., 2016), which supports several pharmacological activities relevant to uterine fibroid management. An ethnopharmacological overview of *G. perpensa* and *A. tanganyicensis*, including their traditional uses, phytochemicals, reported pharmacological activities, potential relevance to fibroids, and ConPhyMP/GA checklist status, is summarized in Table 2.

**TABLE 2:** Ethnopharmacological summary and GA checklist considerations.

Parameter	Gunnera perpensa	Albizia tanganyicensis	Reference(s)
Local name(s)	Ugobho (Zulu, Xhosa)	Umphaphama (Ndebele)	—
Traditional uses	Uterine stimulant, management of retained placenta, menstrual disorders	Used to stimulate libido, relieve menstrual pain, regulate hormonal balance	Simelane et al. (2010)
Reported phytochemicals	Flavonoids, phenolics, alkaloids, tannins, terpenoids, saponins	Steroids, flavonoids, tannins, terpenoids, saponins	Simelane et al. (2010)
Pharmacological activities	Uterotonic, anti-inflammatory, antioxidant, antimicrobial	Analgesic, anti-inflammatory, antioxidant, hormone-modulating	Kaido et al. (1997)
*In vitro* studies	Demonstrated LOX inhibition, protein denaturation inhibition, DPPH antioxidant activity	Limited reports focusing on antioxidant and anti-inflammatory assays	Muleya et al. (2014)
*In vivo* studies	Uterotonic effects demonstrated in animal models	Analgesic effects demonstrated in animal models	Kaido et al. (1997) and Khan et al. (2004)
Relevance to uterine fibroids	May reduce inflammation and oxidative stress contributing to fibroid growth; supports uterine function	May assist *via* hormonal modulation and inflammatory control	Wroclaw Medical University (2023)
ConPhyMP and GA compliance	Moderate–high: taxonomy, traditional use and parts used documented	Moderate: traditional use strong; limited mechanistic and clinical evidence	Heinrich et al. (2022)

*Albizia tanganyicensis* has been associated with toxicity, primarily due to the presence of the neurotoxin 4-methoxy-pyridoxine in its pods (Basson et al., 1970; Steyn et al., 1987). Importantly, this toxicity has not been attributed to the stem bark, which is the focus of our study. In contrast, bark and stem extracts from *A. tanganyicensis* and related *Albizia* species (e.g., *Albizia lebbeck*, *Albizia adianthifolia*) have demonstrated pharmacological potential, particularly anti-inflammatory effects in animal models (Babu et al., 2009). Ethnomedicinal records further report the use of *Albizia* species stem bark for cough, diarrhea, insomnia, irritability, rheumatism, stomach ache, tuberculosis, and wounds (Maroyi, 2018). However, caution is advised due to its potential toxicity, especially in livestock.

It is important to note that the documented cases of toxicity were reported in a different province from where our plant samples were collected. Moreover, our study focuses exclusively on stem bark, not pods, which are the primary source of reported neurotoxicity. This distinction demonstrates the importance of plant part and geographic variation in determining safety and efficacy. Any future therapeutic application of *A. tanganyicensis* should be preceded by rigorous toxicological evaluation to ensure safety, particularly given the documented toxicity of its pods.

## 8 Critical evaluation of the literature

The reviewed literature has consistently reported the ethnomedicinal relevance of *G. perpensa* and *A. tanganyicensis* in managing gynecological conditions, including uterine fibroids. *In vitro* studies on *G. perpensa* demonstrate anti-inflammatory (lipoxygenase [LOX] inhibition, protein denaturation), antioxidant, and uterotonic activities, suggesting pharmacological relevance to uterine fibroid pathology (Kaido et al., 1997; Muleya et al., 2014). Phytochemical screenings for both plants confirmed the presence of bioactive compounds such as flavonoids, phenolics, steroids, and saponins, which are known to exhibit antioxidant and anti-inflammatory effects.

The limitation is that most studies relied on crude extracts without full characterization of active constituents, making it difficult to identify which compounds are responsible for observed effects. The studies also focus on general anti-inflammatory assays without disease-specific models (e.g., human uterine fibroid cells). Data on compound standardization, dose-response relationships, and bioavailability are lacking. The ethnopharmacological relevance is often not directly validated with molecular or clinical data.

### 8.1 Compliance with GA and ConPhyMP standards

*Gunnera perpensa* meets General/Good Assurance (GA) standards and the Consensus Statement on the Phytochemical Characterisation of Medicinal Plants (ConPhyMP) standards more closely due to well-documented traditional preparations and known use in South African ethnomedicine. *Albizia tanganyicensis* requires further taxonomic clarification and clinical substantiation. The documented ethnomedicinal use supports inclusion in ConPhyMP-compliant preclinical investigations. Traditional decoction methods are known for *Gunnera*, less so for *Albizia*, which limits scientific replication. There is a need for benefit-sharing frameworks and informed consent when sourcing knowledge from indigenous communities.

### 8.2 Integration of traditional medicine with modern approaches

Traditional medicine offers valuable leads, particularly in underserved populations where fibroids are prevalent. Combining traditional knowledge with modern pharmacological tools (e.g., liquid chromatography–mass spectrometry (LC–MS), and *in vitro* fibroid cell assays) may enhance drug discovery pipelines. Plant-based uterotonics and hormone modulators may serve as adjunctive or alternative therapies in resource-constrained settings.

## 9 Conclusion

This review highlights the potential relevance of *G. perpensa* and *A. tanganyicensis* in uterine fibroid management, based on their traditional use and preliminary pharmacological findings. *Gunnera perpensa* demonstrates uterotonic, anti-inflammatory, and antioxidant activities in preclinical studies, while *A. tanganyicensis* shows estrogen-modulating and analgesic properties, although current pharmacological evidence is still limited. These biological activities correspond with mechanisms implicated in fibroid pathogenesis and warrant further investigation.

Given the high burden of uterine fibroids among African women and limited accessibility of conventional therapies in many regions, these plants may represent culturally significant and potentially affordable options. However, their therapeutic application remains to be validated in well-designed pharmacological and clinical studies. Future research guided by Good Agricultural Practices (GAP) and the Consensus on Phytomedicine Methodological Practices (ConPhyMP) framework will be critical to ensure methodological rigor, reproducibility, and ethical integration of traditional remedies into modern healthcare, thereby contributing to more inclusive approaches in women’s health.
